# SP3 Protocol for Proteomic Plant Sample Preparation Prior LC-MS/MS

**DOI:** 10.3389/fpls.2021.635550

**Published:** 2021-03-10

**Authors:** Kamil Mikulášek, Hana Konečná, David Potěšil, Renata Holánková, Jan Havliš, Zbyněk Zdráhal

**Affiliations:** ^1^Mendel Centre for Plant Genomics and Proteomics, Central European Institute of Technology, Masaryk University, Brno, Czechia; ^2^National Centre for Biomolecular Research, Faculty of Science, Masaryk University, Brno, Czechia

**Keywords:** bottom-up, protein cleanup, *Arabidopsis thaliana*, sodium dodecyl sulfate removal, single-pot solid-phase-enhanced sample preparation, carboxylated magnetic beads, filter-aided sample preparation, mass spectrometry

## Abstract

Quantitative protein extraction from biological samples, as well as contaminants removal before LC-MS/MS, is fundamental for the successful bottom-up proteomic analysis. Four sample preparation methods, including the filter-aided sample preparation (FASP), two single-pot solid-phase-enhanced sample preparations (SP3) on carboxylated or HILIC paramagnetic beads, and protein suspension trapping method (S-Trap) were evaluated for SDS removal and protein digestion from *Arabidopsis thaliana* (AT) lysate. Finally, the optimized carboxylated SP3 workflow was benchmarked closely against the routine FASP. Ultimately, LC-MS/MS analyses revealed that regarding the number of identifications, number of missed cleavages, proteome coverage, repeatability, reduction of handling time, and cost per assay, the SP3 on carboxylated magnetic particles proved to be the best alternative for SDS and other contaminants removal from plant sample lysate. A robust and efficient 2-h SP3 protocol for a wide range of protein input is presented, benefiting from no need to adjust the amount of beads, binding and rinsing conditions, or digestion parameters.

## Introduction

The efficient sample preparation for proteomic analysis of plants represents a real challenge. Plants contain a low concentration of proteins and high levels of secondary metabolites potentially interfering with proteome analysis (Hussein and El-Anssary, 2018). Harsh conditions, usually mechanical force or highly effective sonication, have to be applied to disrupt cell walls. These procedures are often followed by traditional protein extraction protocols (TCA/acetone precipitation or phenol extraction) to concentrate proteins and avoid protein degradation caused by abundant proteases. Since precipitated proteins are often difficult to resolubilize, the extraction protocols frequently contain chaotropes and surfactants (Komatsu, 2008; Wang et al., 2008; Takáč et al., 2017; Niu et al., 2018; Jorrin-Novo et al., 2019). Plant proteomics currently has moved from traditional gel-based strategies in which many low abundant, extreme pI and hydrophobic proteins were underrepresented, to gel-free shotgun workflows. Consequently, sample preparation approaches containing the removal of substances interfering with digestion and MS analysis are of great importance (Song et al., 2018; Wang et al., 2018).

Several new strategies for proteome sample preparation enabling an efficient and robust bottom-up analysis were introduced in recent years. Many protocols were based on whole-lysate protein solubilization and denaturation by sodium dodecyl sulfate (SDS). However, since the application of SDS may have also negative consequences, suppressing both MS signal and protease activity (Min et al., 2015; Scheerlinck et al., 2015), SDS has to be depleted before digestion and MS analysis. Traditional approaches to remove surfactants and other contaminants (chaotropes, salts, buffers, solvents, and tags) prior MS analysis were reviewed elsewhere (Feist and Hummon, 2015; Tubaon et al., 2017). More recently, several innovative attempts to get rid of contaminants were proposed, including the most widely adopted filter-aided sample preparation (FASP), the relatively new single-pot solid-phase-enhanced sample preparation (SP3), and the protein suspension trapping (S-Trap).

In the FASP workflow, the sample lysate is applied to the centrifugal 30 kDa cut-off ultrafiltration unit. Low-mass contaminants are washed out and proteins are then digested on-membrane. In-solution retained peptides are ready for the MS analysis (Wiśniewski et al., 2009, 2011). Over the years, various useful modifications of the classical FASP protocol were published (Erde et al., 2014, 2017; Huber et al., 2014; Glatter et al., 2015; Nel et al., 2015; Lipecka et al., 2016; Li et al., 2017; Ni et al., 2017; Potriquet et al., 2017; Wiśniewski, 2017, 2018, 2019; Zhang et al., 2020). However, the FASP workflows are expensive and rather time-consuming. Moreover, traces of remaining SDS are frequently detected after FASP and they have to be removed by additional extraction into ethyl acetate (Yeung and Stanley, 2010). Only a few studies used FASP strategies for plant samples, e.g., the *Arabidopsis thaliana* leaf (Song et al., 2018), the barley leaf (Wang et al., 2018), and the maize leaf (Balliau et al., 2018).

As alternatives to traditional FASP protocols, less laborious and faster single-vessel strategies were utilized recently for sample preparation which included removal of SDS and other substances interfering with digestion and MS analysis. Krijgsveld and his team introduced the innovative SP3 method based on an efficient and fast nonselective binding of proteins on paramagnetic beads utilizing Sera-Mag Carboxylate-Modified magnetic beads (Hughes et al., 2014). Proteins immobilized on beads are separated from common contaminants and enzymatic digestion is realized directly on beads. MS analysis of in-solution retained peptides can consequently proceed without any additional cleanup. Since this pioneering approach, more detailed studies on SP3 protocol-specific conditions were published (Moggridge et al., 2018; Dagley et al., 2019). Hughes et al. (2019) summarized the most critical requirements for maximum performance of the SP3 procedure, including the beads/protein ratio, the working concentration of beads in the sample, and on-bead digestion conditions. For the automated SP3 technology, Müller (Müller et al., 2020) adapted a liquid handling robot in 96-well format suitable for low input clinical samples, starting from 100 HeLa cells. As an alternative to carboxylated beads, the mixed-mode hydrophilic interaction chromatography on magnetic microparticles MagReSyn HILIC (ReSyn Biosciences) was also extensively studied (Hughes et al., 2014; Stoychev et al., 2017, 2018). Another single-vessel methodology tested successfully for contaminants removal was based on trapping of protein suspension in the quartz filter of the spin column S-Trap (ProtiFi). Contaminants could be efficiently removed in the flowthrough, the protein suspension also facilitated protease digestion (Zougman et al., 2014; HaileMariam et al., 2018; Ludwig et al., 2018).

The presented single-vessel sample preparations were benchmarked mostly for analysis of human and animal tissues, only several studies dealt with complex plant samples. No sample preparation method was found to be universally applicable for all sample types. Song et al. (2018) evaluated three sample preparation protocols for *A. thaliana* green leaves based on urea solubilization, methanol/chloroform extraction, or phenol-based extraction. All methods were improved when combined with FASP. Wang et al. (2018) analyzed barley leaves and compared two FASP protocols using either SDS or sodium deoxycholate and three in-solution protocols. Two spin filter-based protocols provided a higher efficiency than the other protocols. Lewandowska et al. (2019) was not very successful in applying SP3 Carboxy or SP3 HILIC to barley anthers. No SP3 clean-up study on green plant leaves was published to date.

As mentioned above, most of the comprehensive benchmarking dealt with proteins of human and animal origins. The following studies showed rather diverse results: Sielaff et al. (2017) evaluated protocols on carboxylate-modified beads, FASP, and in-StageTip (iST) for HeLa cell proteins. All three workflows showed similar results for 20 μg of protein. Unlike FASP, both SP3 and iST provided high proteome coverage even at 1 μg level. Stoychev et al. (2017) studied colon carcinoma cell extract; the HILIC SP3 workflow provided an approximately two-fold increase in peptide recovery and over 30% increase in identified post-translational modifications of peptides and unique proteins, compared to FASP or SP3 on carboxylated beads. Moggridge et al. (2018) found SP3 performance on HILIC beads vs. carboxylated beads comparable for HEK cell lysate; carboxylated beads exhibited significant cost savings per assay. Ludwig et al. (2018) evaluated S-Trap, FASP, and in-solution digest for colorectal cancer SW480 cell lysate. S-Traps outperformed other methods regardless of lysis conditions. HaileMariam et al. (2018) tested S-trap (single or 96-well filter plate) and FASP for bacterial whole cell lysate and human sputum; both yielded similar results regarding protein and peptide identifications. Dagley et al. (2019) compared SP3 and FASP for HeLa lysate; both methods detected comparable numbers of peptides. Gonzalez-Lozano et al. (2019) combined an immunoprecipitation protocol for mouse brain proteins with FASP purification or SP3 capture on carboxylated beads; no major differences in identifications were found. As documented above, particular clean-up strategies might have an ambiguous impact on samples of different origins.

In this study, we evaluated four sample processing methods applied to the model plant *A. thaliana*; whole leaf lysates are processed by four workflows: the classical FASP, two SP3 protocols using carboxylated or HILIC paramagnetic beads, and the S-Trap protocol. Proteome analysis performance evaluation, as well as the time needed for sample preparation and cost per sample, indicate that the SP3 protocol on carboxylated beads can be selected as the preferred method. Finally, the robust optimized SP3 protocol applicable for a wide range of input protein amounts is presented, and its performance is benchmarked against the FASP used routinely in our lab. Thoroughly optimized SP3 workflow represents the potential to outperform other protein sample preparation strategies especially for low protein amounts and might provide a solid platform for broad proteomic applications not only in plants.

## Materials and Methods

### Plant Material and Growth Conditions

*Arabidopsis thaliana* ecotype Columbia 0 plants were grown on soil, 3 weeks after planting rosette leaves were harvested and ground in liquid nitrogen in the Freezer/Mill 6870 (SPEX SamplePrep) in three cycles of 2 min grinding and 2 min cooling. Fine plant powder was stored at −80°C.

### Protein Extraction

SDT Lysis: Aliquot of 1 g of frozen AT plant tissue powder was solubilized in Thermo Mixer C (Eppendorf) in 1 ml of hot SDT buffer (4% SDS, 100 mM DTT; 100 mM Tris-HCl, pH 7.6) for 2 h at 95°C and 1,000 rpm. To remove insoluble material from the sample, the extract was centrifuged at room temperature (RT) for 10 min and 20,000 g.

### Tryptophan Fluorescence Assay

For protein and peptide quantification, a sensitive assay based on the fluorescence spectrometry of tryptophan in a buffer containing 8 M urea (Wiśniewski and Gaugaz, 2015) was performed on the Cary Eclipse Fluorescence Spectrophotometer (Agilent Technologies). The assay (unlike common colorimetric protein assays) is fully compatible with substances typically used for tissue lysis, such as surfactants or reducing agents. The experiment recoveries were calculated as the ratio of corresponding amounts of resulting peptides after digestion and the input protein amounts obtained based on tryptophan fluorescence measurements.

### Alkylation and Quenching

Alkylation and quenching of proteins for SP3 HILIC, SP3 Carboxy, and S-Trap protocols were originally done according to Moggridge et al. (2018). In the final SP3 carboxylated beads protocols, iodoacetamide (IAA) was added to a final concentration of 20 mM, and mixtures were incubated at RT for 30 min in the dark, Thermo Mixer, 600 rpm at 24°C. Samples were quenched by the addition of dithiothreitol (DTT) to a final concentration of 5 mM DTT.

### Filter-Aided Sample Preparation

Microcon-30 kDa centrifugal filter concentrators MRCF0R030 (for the protein loads 0.1–100 μg), and Amicon Ultra-4 centrifugal filter unit UFC803096 (for the 5 mg protein load) were purchased from Merck. In the previously established FASP procedure described by Wiśniewski (2019), extracted proteins were transferred to centrifugal filter units, washed with 8 M urea, and then with 50 mM ammonium bicarbonate by centrifugation at 14,000 g. Proteins were digested with trypsin, enzyme/protein 1:50 (or 1:100 for 5 mg load), and incubated overnight at 37°C. Due to incomplete removal of SDS (traces were observed during MS analysis occasionally), the tryptic peptides were always extracted into the water-immiscible ethyl acetate, according to Yeung and Stanley (2010).

### SP3 Carboxy

Sera-Mag Carboxylate-Modified Magnetic Beads and *SpeedBeads* (GE Life Sciences) have free carboxyl groups on the surface for the covalent coupling of target molecules. One layer (Sera-Mag Beads) or two layers (Sera-Mag SpeedBeads) of magnetite are coated onto the core of beads. Sera-Mag Beads Carboxylate-Modified hydrophilic solids (GE Life Sciences), cat. no. 24152105050250, were combined 1:1 with hydrophobic solids, cat. no. 44152105050250. A detailed step-by-step version of the Basic SP3 Protocol for protein cleanup processing a protein load in a range of 0.1–100 μg is available in the Supplementary Material. Sera-Mag SpeedBeads Carboxylate-Modified hydrophilic solids (GE Life Sciences), cat. no. 45152105050250, were 1:1 combined with hydrophobic solids, cat. no. 65152105050250. A detailed step-by-step version of the Large-Scale SP3 Protocol for protein cleanup processing a protein load up to 10 mg is available in the Supplementary Material.

### SP3 HILIC

Magnetic multi-mode HILIC microparticles MagReSyn HILIC (ReSyn Biosciences) were used according to the manufacturer’s instructions (Product Instruction Guide MagReSyn HILIC) and the RAPOBD protocol (*Protocol for cleanup of reduced and alkylated proteins with on-bead digestion*). A detailed version of the protocol is available in the Supplementary Material.

### S-Trap

S-Trap Mini Spin Columns (ProtiFi) were used according to slightly modified manufacturer’s instructions (*S-Trap Mini Spin Column Digestion Protocol 3.6*). A detailed version of the protocol is available in the Supplementary Material.

### Protein Digestion

Sequence Grade Modified Trypsin was purchased from Promega. The digestion of purified protein samples proceeded in 50 mM ammonium bicarbonate (AB) at 37°C and 1,000 rpm using the Thermo Mixer C. Digestion times are indicated at individual experiments and in the final protocols.

### Peptide Quality Control After FASP and SP3 Carboxy Protocols

The initial peptide mixture quality control and concentration measurements before FASP and SP3 Carboxy methods benchmarking test and for SP3 Carboxy protocol optimizing were carried out by Ultimate 3000 RSLCnano system (Thermo Fisher Scientific, Waltham, MA, United States) on-line connected to UV-Vis detector and ion trap mass spectrometer HCT Ultra (Bruker Daltonics, Bremen, Germany). The processed peptide mixture was separated onto C18 stationary phase (Acclaim PepMap C18, 2 μm particles, 75 μm × 150 mm) by following mobile phases (A: 0.1% FA in water; B: 0.1% FA in 80% acetonitrile, flow rate 300 nl/min) and the gradient: the elution started at 2% (0–4 min) of mobile phase B, increased from 2 to 60% (4–44 min), then increased to 80% during 1 min and remained at this state for the next 5 min. The peptide concentration of samples were derived from MEC1 cells calibration curve. The calibration curve consists of five calibration points and covers a range of 10–500 ng. Based on these findings the appropriate sample volume corresponding to 2 μg of peptide mixture was injected during final LC-MS/MS analysis.

### LC-MS/MS Analysis – Four Sample Preparation Methods Comparison, SP3 Carboxy Protocol Adjustment

Ultimate 3000 RSLCnano system on-line connected to Orbitrap Elite hybrid spectrometer (Thermo Fisher Scientific, Waltham, MA, United States) was utilized to method assessment analysis and for optimizing of SP3 Carboxy protocol. Prior to LC separation, tryptic digests were on-line concentrated and desalted using trapping column (100 μm × 30 mm, 40°C) filled with 3.5-μm X-Bridge BEH 130 C18 sorbent (Waters). After washing of trapping column with 0.1% formic acid (FA), the peptides were eluted from the trapping column onto an analytical column (Acclaim Pepmap100 C18, 3 μm particles, 75 μm × 500 mm, 40°C; Thermo Fisher Scientific) and separated by 120 min nonlinear gradient program (mobile phase A: 0.1% FA in water; mobile phase B: 0.1% FA in 80% acetonitrile). Equilibration of the trapping column and the column was done prior to sample injection to sample loop. The analytical column outlet was directly connected to the Digital PicoView 550 (New Objective) ion source with sheath gas option and SilicaTip emitter (New Objective; FS360-20-15-N-20-C12) utilization. Active Background Ion Reduction Device (ABIRD, ESI Source Solutions) was installed. MS data were acquired in a data-dependent strategy selecting up to top 10 precursors based on precursor abundance in the survey scan (*m/z* 350–2,000). The resolution of the survey scan was 60,000 (at *m/z* 400) with a target value of 1 × 10^6^ ions, one Microscan and maximum injection time of 1,000 ms. HCD MS/MS spectra were acquired with a target value of 50,000 and a resolution of 15,000 (at *m/z* 400). The maximum injection time for MS/MS was 500 ms. Dynamic exclusion was enabled for 45 s after one MS/MS spectra acquisition. The isolation window for MS/MS fragmentation was set to 2 *m/z*.

### LC-MS/MS Analysis – FASP and SP3 Carboxy Benchmarking Test

To measure a final dataset following material and methods were used. Mass spectrometry analysis of the peptide mixture was done using RSLCnano system (Thermo Fisher Scientific, Waltham, MA, United States) on-line connected to Orbitrap Q-Exactive HF-X system (Thermo Fisher Scientific, MA, United States). Prior to LC separation, tryptic digests were concentrated and desalted on-line using a cartridge trapping column (300 μm × 5 mm) filled with 5-μm particles C18 PepMap100 sorbent (Thermo Fisher Scientific, Waltham, MA, United States). The peptides were eluted from the trapping column onto an Acclaim Pepmap100 C18 analytical column (3-μm particles, 75 μm × 500 mm; Thermo Fisher Scientific) and separated by the following gradient program (mobile phase A: 0.1% FA in water; mobile phase B: 0.1% FA in 80% acetonitrile, flow rate 300 nl/min). The gradient elution started at 2% (0–5 min) of mobile phase B, increased from 2 to 35% (5–107 min), then increased linearly to 80% (107–115 min) of mobile phase B and remained at this state for the next 5 min. Equilibration of the trapping column and the column was done prior to sample injection to sample loop. For a quality control purpose, Biognosys iRT peptides were added during each analysis. The analytical column outlet was directly linked to the Digital PicoView 550 (New Objective) ion source with sheath gas option and SilicaTip emitter (New Objective; FS360-20-15-N-20-C12) utilization. ABIRD (ESI Source Solutions) was installed. MS data were acquired in a data-dependent strategy selecting up to top 20 precursors based on precursor abundance in the survey scan (*m/z* 350–2,000). The resolution of the survey scan was 120,000 (at *m/z* 200) with a target value of 3 × 10^6^ ions and a maximum injection time of 100 ms. HCD MS/MS spectra were acquired with a target value of 5 × 10^4^ and resolution of 15,000 (at *m/z* 200). The maximum injection time for MS/MS was 50 ms. Dynamic exclusion was enabled for 40 s after one MS/MS spectra acquisition. The isolation window for MS/MS fragmentation was set to 1.2 *m/z*.

### Protein Identification and Label-Free Protein Quantification

Processing of RAW data files was performed using Maxquant (Cox and Mann, 2008, MQ) proteomics platform (v. 1.6.10.43) with a built-in Andromeda search engine (Cox et al., 2011). Data were searched against Uniprot *A. thaliana* proteome (version from 20190703) and MQ contaminant database. Potential contaminants, reverse sequences, and proteins identified by only peptide modification site were filtered out from the final protein group list. The first search peptide tolerance was set to 20 ppm. For the main search, 4.5 and 20 ppm were set for precursor and fragment ions as limited values. Individual peptide mass tolerance was applied. Carbamidomethylation of cysteine was set as a fixed modification, while oxidation (M) and deamidation (N, Q) were set as a variable. Trypsin was used as the protein-cleaving enzyme with one and two missed cleavages. Only peptides and proteins with false discovery rates (FDRs; *q*-values) < 1% were considered for final data evaluation. Quality control analysis was done *via* PTXQC tool (Bielow et al., 2016; v.1.0.1). Consecutive bioinformatics analysis was realized using Perseus (Tyanova and Cox, 2018; v. 1.6.10.50). The mass spectrometry proteomics data have been deposited to the ProteomeXchange Consortium *via* the PRIDE (Perez-Riverol et al., 2019) partner repository with the dataset identifier PXD022688.

### Gene Ontology Analysis

The protein classification and mapping according to cellular components (GOCC, gene ontology cellular component) were done *via* Perseus software (v. 1.6.10.50). *Arabidopsis thaliana* protein annotations were downloaded from https://datashare.biochem.mpg.de/s/qe1IqcKbz2j2Ruf. For annotations, protein group identity was summarized for each repetition at the protein level. The GOCC frequency for each expression was counted and a relative content in the samples were calculated. Data were sorted in descending order.

## Results

As the first step, we selected four established methods and compared their performance for processing of typical plant protein samples, SDS-containing protein extracts of *A. thaliana* leaves. Then, we focused on the two most perspective techniques. We optimized the SP3 technique for a wide range of plant protein loads and finally, we compared the performance of our SP3 protocols with the FASP technique which was taken as a benchmark.

### Four Sample Preparation Methods Comparison

The initial comparison of sample preparation for bottom-up proteomics utilizing 60 μg of AT total protein load was based on the previously described methods: SP3 on carboxylated beads (Hughes et al., 2014; Moggridge et al., 2018), SP3 on HILIC beads (Protocol for cleanup of reduced and alkylated proteins with on-bead digestion, Retrieved from http://www.resynbio.com/hilic), S-Trap (Zougman et al., 2014), and FASP (Wiśniewski et al., 2009). In this case, we applied recommended protocols for all four methods. Regarding digestion conditions, we evaluated consecutive digestion with trypsin in our experiment as decreasing activity of the modified trypsin with blocked autolysis was reported being in solution over time (Finehout et al., 2005) and multienzyme digestion was successfully applied on protein extracts mostly from mammalian cell cultures (Wiśniewski and Mann, 2012). As opposed to the reported positive effects of multienzyme digestion we found no substantial differences comparing overnight digestion with trypsin at 37°C and digestion with a mixture of trypsin/LysC under the same conditions in case of our plant material (data not shown) and we did not explore the multienzyme option further. In case of consecutive digestion, the first digestion step with 6 μg trypsin was followed by the second step, when the remaining proteins were subsequently cleaved with the fresh load of 6 μg trypsin. The initial protease:protein ratio 1:10 was increased to 1:5 after the second enzyme addition. All the samples were processed in triplicates.

**Figure 1:**
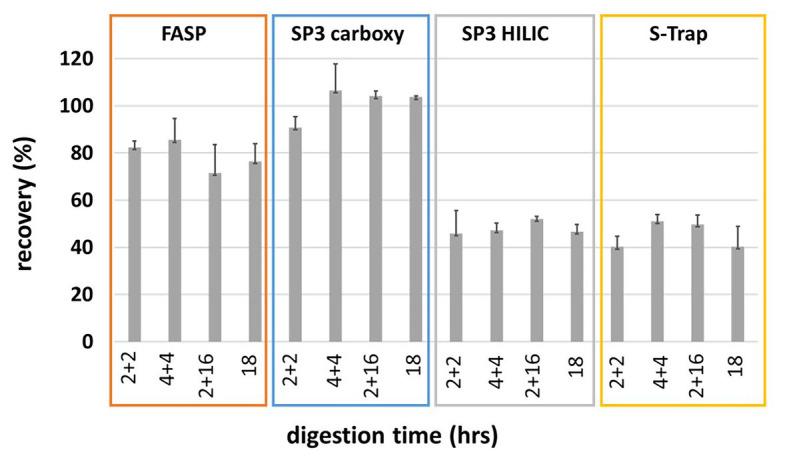
Peptide recoveries based on peptide concentration obtained by tryptophan assay. The recoveries are not corrected to trypsin addition.

We tested a total of 16 combinations: four sample preparation workflows (SP3 on carboxylated beads, SP3 on HILIC beads, S-Trap, and FASP) in various total digestion times (4, 8, and 18 h), three of them represented by double addition of trypsin: 2 + 2, 4 + 4, 2 + 16 h of incubation at 37°C. The results were compared to the standard uninterrupted overnight digestion for 18 h. Initially, we measured final peptide amounts *via* the tryptophan assay and calculated the recovery of individual methods under given digestion conditions (Figure 1). The peptide recovery of the SP3 Carboxy method clearly outperformed SP3 HILIC and S-Trap methods. The FASP technique was the second-best providing 10–20% lower recoveries compared to SP3 Carboxy method. The recovery values are not corrected for the input amount of trypsin (12 or 6 μg), therefore they should be taken only for comparative purposes.

Next, we evaluated the influence of the total incubation time and/or addition of trypsin on digestion efficiency based on the results of LC-MS/MS analysis. The relative content of missed cleavages was calculated as a mean from triplicate of all identified peptide sequences, as well as a proportion of summed peptide intensity (Supplementary Figure S1) showing proportion of peptides with one miscleavage in the range of 10–18 and 7–15%, respectively, over all methods under given conditions. The lowest specificity revealed the SP3 HILIC method (15–18%) but in general, we observed no significant differences. Based on these results we selected the 18 h digestion without any further trypsin addition and the trypsin:protein ratio 1:10 as a reference condition for further data analysis.

Under these digestion conditions, we compared the number of identified protein groups and individual method variability.

**Figure 2:**
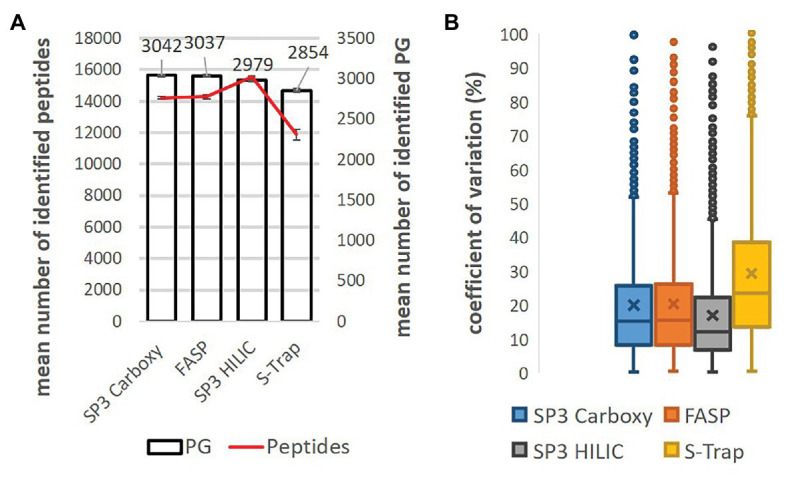
Comparison of SP3 Carboxy, FASP, SP3 HILIC, and S-Trap performance (18 h digestion, trypsin:protein 1:10, three replicates). **(A)** Mean number of identified protein groups (bar chart) and peptides (line chart). Technical variability of protein group and peptide detection is displayed as error bars. **(B)** Coefficients of variation of protein group intensity (%). Only shared protein groups (*n* = 1986) across the whole dataset were considered. Median normalization was applied [data were log_(2)_ transformed].

Concerning the number of identified protein groups and peptides, FASP and SP3 Carboxy provided almost identical average numbers (Figure 2A; Supplementary Table 1). SP3 HILIC provided a slightly lower average number of identified protein groups but the highest number of identified peptides. The lowest average number of identified protein groups was obtained by S-Trap. It is likely related to insufficient SDS removal which we were not able to improve.

To estimate the overall correlation between individual methods, the coefficient of determination (*R^2^*) was assessed for the samples digested for 18 h (Supplementary Figure S2). The injected amount was adjusted based on the previous quality control analysis and the median normalization was applied to eliminate systematic bias derived from non-biological sources. Median protein group intensity of three replicates and only shared protein groups (*n* = 1986) across the whole dataset were considered for calculations. The highest correlation (*R^2^* = 0.88) was observed between FASP and SP3 Carboxy methods, on the other side the lowest correlation (*R^2^* = 0.73) was between S-Trap and SP3 HILIC methods. To assess, the technical variability of each method (three replicates) the boxplots for the coefficients of variation (CV) were calculated (Figure 2B). The lowest median CV (12.1%) was displayed by SP3 HILIC method, SP3 Carboxy (15.3%) and FASP (15.5%) were almost in line and the highest variability (CV = 23.4%) was obtained by the S-Trap method.

In summary, SP3 Carboxy and FASP and SP3 HILIC provided the comparable performance in case of *A. thaliana* protein SDS-containing extracts. Moreover, the presented SP3 approach with carboxylated beads demonstrated several benefits – easy handling, short working time, and lower cost in comparison with other tested methods. Sera-Mag beads cost per sample is more than ten times lower than the cost of FASP, HILIC beads, or S-Trap columns. Encouraged by the benchmarking results, we selected for processing plant material the SP3 workflow with carboxylated magnetic beads for further optimization followed by a comprehensive comparison with the FASP method. Based on low peptide recoveries (see Figure 1) SP3 HILIC and S-Trap protocols were not selected for further optimization.

### SP3 Carboxy Protocol Adjustment

Although recently published SP3 protocols using carboxylated beads were already optimized extensively (Moggridge et al., 2018; Dagley et al., 2019; Hughes et al., 2019), yet they unanimously called for adjusting selected parameters (e.g., amount of beads or trypsin) to particular input of protein. Even though our protocol was particularly designed according to the recommendations, the workflow was reasonably modified with aim to guarantee method feasibility for a broad protein range with no need to adjust the amounts of beads or trypsin to protein input. We optimized the SP3 Carboxy protocol for two representative total protein input amounts: 1 μg and 10 mg in SDS-containing protein extract (SDT buffer).

#### Basic Protocol – Up to 100 μg Protein Inputs

**Figure 3:**
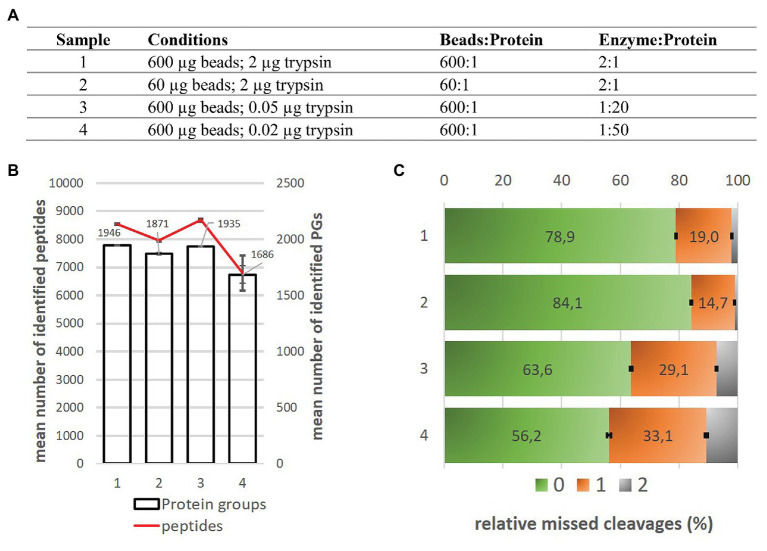
Comparison of SP3 Carboxy method performance for 1 μg input amount of *Arabidopsis thaliana* samples. **(A)** The overview of conditions applied in this experiment. **(B)** Mean number of protein groups and peptides identified under given conditions. Technical variability of protein groups and peptide detection is displayed as error bars for three replicates. **(C)** Relative missed cleavages content (%) corresponds to the number of identified peptides without any missed cleavage (green), with one (orange) and two (gray) missed cleavages. Data were calculated as an average value from three replicates. Standard deviation was applied for error bars calculation.

In case of the Basic Protocol, we focused on SP3 Carboxy performance for low input amounts and we selected protein input of 1 μg. Next to the previously recommended parameters (Hughes et al., 2019) concerning bead:protein ratio, working volumes, and trypsin:protein ratio, we reduced bead:protein ratio 10 times and tested different trypsin:protein ratios as well (Figure 3A). All other parameters used for this experiment (working volumes, etc.) could be found in the Basic Protocol (see Supplementary Material). The results indicate that for low microgram amounts of protein a high excess of beads is not harmful; it is even preferred for satisfactory results. In opposite, the reduction of bead:protein ratio 10 times resulted in a drop of mean number of identified proteins (Figure 3B). Similarly, the high excess of trypsin (enzyme:protein ratio 2:1) was beneficial for the digestion of the low protein amount. The reducing of trypsin:protein ratio led to the decreased number of identified proteins/peptides and the increased number of peptides with one or more miscleavages (above 30% for ratio 1:50, Figure 3C).

As no negative effect of massive bead excess for detection of low microgram loads was observed we selected 600 μg of beads and 2 μg of trypsin as a compromise in regard to bead:protein and trypsin:protein ratios even for wider protein input range, and used them for the final experiment of processing of three-order of magnitude range of protein inputs (0.1–100 μg) which corresponds to protein content of majority of protein samples processed in our laboratory.

#### Large-Scale Protocol – mg Protein Inputs

**Figure 4:**
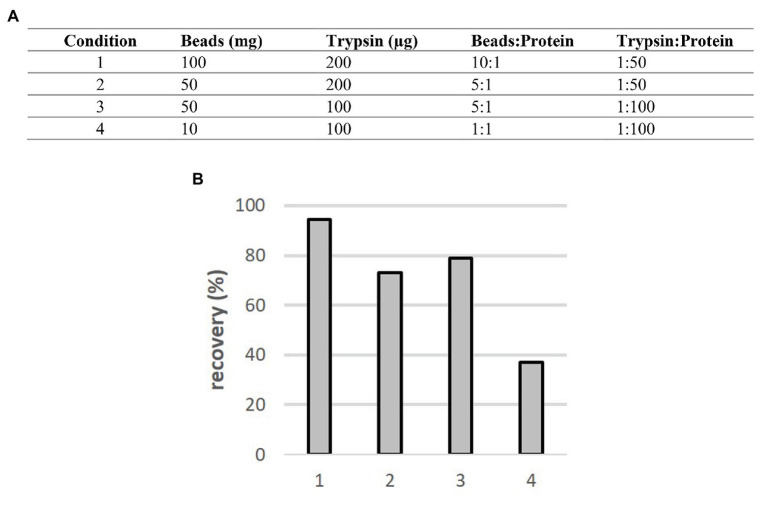
Comparison of SP3 Carboxy method performance for 10 mg input amount of *A. thaliana* samples. **(A)** The overview of conditions applied in this experiment. **(B)** Obtained recoveries (including the first washing step) for individual conditions.

As an enormous amount of input protein material may be required for downstream processing prior LC-MS/MS (e.g., for the immunoprecipitation enrichment; acetylome analysis; Mo et al., 2015; Lv et al., 2016; Hartl et al., 2017; Basisty et al., 2018; Liu et al., 2019) 10 mg of *A. thaliana* total protein sample was used for testing of SP3 Carboxy protocol. Again, the SP3 method was directly employed without any previous protein precipitation step using SDS-containing protein extract. This high amount of protein places an increased demand on the efficiency of protein binding step, as well as quantitative peptide release from beads. In this experiment, three ratios of beads:protein and two trypsin:protein ratios were tested to assess proper digestion and quantitative peptide release from beads (Figure 4). The highest peptide recovery we obtained using beads:protein ratio 10:1 (higher ratios were not tested with respect to beads consumption). As expected, peptide yields were decreased with lower beads:protein ratios (in case of ratio 1:1 by a factor of 2.5 compared to ratio 10:1). The trypsin load of 200 μg had a negligible effect compared to 100 μg. To assess possible sample losses, we added the additional 50 mM ammonium bicarbonate washing steps after collection of digest solution from beads. Detailed protocol with all other parameters used for this experiment (working volumes, etc.) could be found in the Large-Scale Protocol (see Supplementary Material).

Peptide amounts in the first washing step represented up to 12% of original input (Supplementary Figure S3). The peptide amount in the second additional washing step was rather low (<3%). Thus, the pooling of digest and the first wash will improve qualitative and quantitative assessment of the high-load samples and it was implemented in the protocol.

In summary, these results proved the capability of the SP3 Carboxy method for processing of high-load protein samples up to 10 mg, suggesting ratios beads:protein 10:1 and trypsin:protein 1:100 for an efficient processing including combination of one wash of beads with the original digestion supernatant as mentioned above.

### FASP and SP3 Carboxy Methods Dynamic Range Benchmarking Test

We compared the proposed SP3 Protocols utilizing carboxylated magnetic beads (see Supplementary Material) to the established FASP procedure used routinely in our laboratory. Both methods were tested for the range of 0.1–100 μg of AT protein (Basic SP3) and also for 5 mg of AT protein (Large-Scale SP3). The samples were analyzed by LC-MS/MS in triplicates. Method repeatability was evaluated by pentaplicate measurements for 100 μg protein input. The samples were analyzed in increasing protein load order. The only half of the final digested sample was injected for 0.1 and 1 μg input amounts to prevent loss of sample from technical issues. In case of higher sample loads (≥10 μg), we injected about 2 μg of tryptic peptide mixture only to reduce detector saturation.

**Figure 5:**
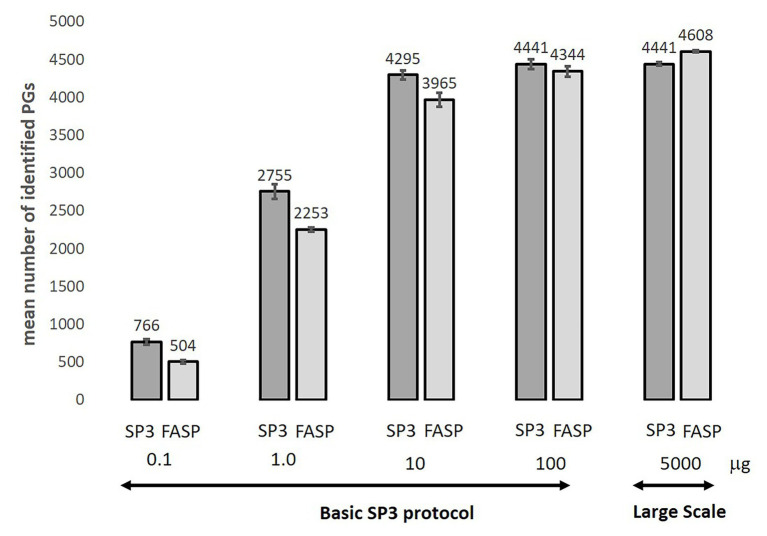
Comparison of mean number of identified protein groups for SP3 and FASP methods over-tested protein load range. Standard deviation was applied for error bars calculation. Data were calculated as the average value from 3 or 5 (100 μg) replicates. The only half of the final digested sample was injected for 0.1–1 μg input amounts to prevent loss of sample from technical issues. In case of higher sample loads (≥10 μg), we injected about 2 μg of tryptic peptide mixture.

A significant increase was observed in the mean number of PGs per replicate and number of unique PGs (sum per all replicates) identified for the three lowest protein loads (0.1, 1, and 10 μg) using the SP3 Carboxy method. Mean number of identified protein groups was 766 (1,018 unique protein groups) using SP3 Carboxy compared to 504 (690) by FASP in case of 0.1 μg protein load and 2,755 (3,032) PGs compared to 2,253 (2,703) PGs identified by SP3 Carboxy and FASP, respectively, in case of 1 μg load. The mean number and number of unique protein groups were not significantly different for 100 μg protein loads between the methods. The mean number of identified PGs was about 4% lower in case of 5 mg protein input using Large-Scale SP3 documenting feasibility of utilization of Large-Scale Protocol (Figure 5; Supplementary Figure S4).

**Figure 6:**
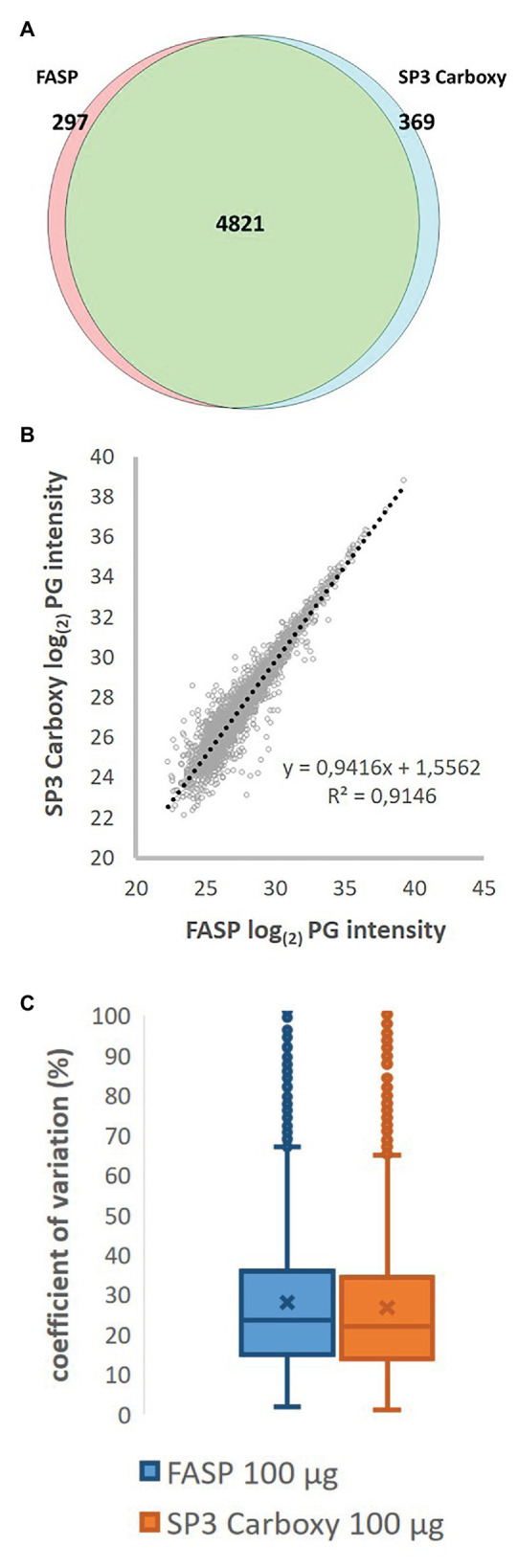
Qualitative and quantitative comparison of FASP and Basic SP3 protocol (100 μg protein input, pentaplicate). **(A)** Overlap of identified unique protein groups. **(B)** Scatter plot of log2 transformed intensities of shared PGs. Median intensity and median normalization approach were employed to calculate protein group intensity. **(C)** Coefficient of variation of shared PGs intensities.

The 3% coefficient of variation for mean number of identified protein groups at 100 μg load was detected for both methods, with overall 9% maximal CV for number of identified protein groups over-tested range. Detected proteins covered over six intensity orders. The Venn diagram shows 88% intersection of unique PGs identified in pentaplicate measurement for SP3 and FASP methods (Figure 6A). Both datasets shared (4,821) protein groups (totally identified 5,487 PGs). It corresponds to 22,371 shared peptides which represent over 52% of all identified peptide sequences for both methods (42,903 unique peptides altogether; function match between run not used).

Similar performance of both methods at a quantitative level is indicated by the high value of the coefficient of determination, exceeding 0.91, calculated based on intensities of identified protein groups detected at all 10 samples (3,073 PGs). To estimate the method repeatability, CV for protein group intensity between both methods was employed. After median normalization, we obtained the median CV 22.12 and 23.53% for SP3 Carboxy (Basic SP3 Protocol) and FASP methods, respectively (Figures 6B,C). Altogether, both methods showed a high quantitative repeatability among five technical replicates for 100 μg protein load.

**Figure 7:**
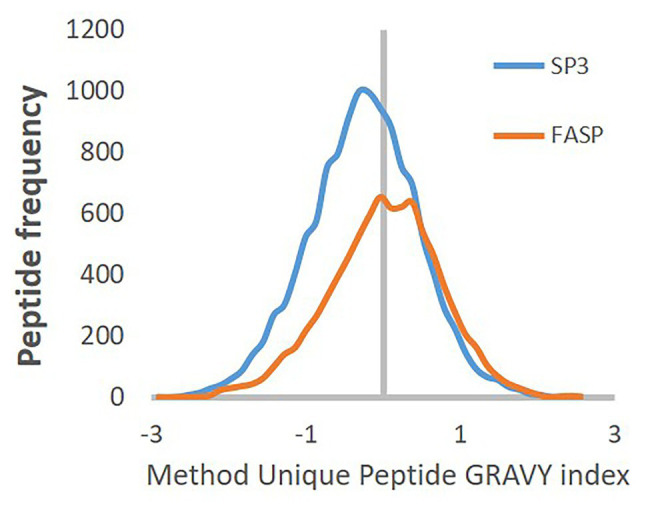
GRAVY index distribution of peptide uniquely identified by FASP (orange) or SP3 Carboxy (blue) method.

To examine systematic bias concerning the physicochemical properties of peptides uniquely identified in the different preparation methods, the protein and peptide molecular mass distributions, and GRAVY index were investigated. Closer inspection of the first two mentioned features showed minimal systematic bias between both methods. However, the peptide GRAVY index indicates a subtle shift of SP3 peptide distribution to more hydrophilic values, mainly visible in case of peptides uniquely detected by individual method (Figure 7; Supplementary Figures S5, S6).

Finally, we compared both methods in terms of the biological features of identified proteins. GO analysis did not reveal any method preference for any protein class. We observed a higher relative content of apoplast and cell wall protein groups for 0.1 μg loads in case of both methods (Supplementary Figure S7). Differently, lower relative content was discovered for endoplasmic reticulum proteins, the integral component of membrane proteins, and proteins of the extracellular region. No differences for higher loads were observed. This discrepancy could be attributed to lower coverage of low input samples. The content of non-annotated protein groups was under 8.20%.

## Discussion

In summary, we demonstrated applicability of SP3 method for processing SDS containing leave lysates of *A. thaliana* without necessity of follow-up cleanup for a wide range of protein inputs. We proposed two modified protocols. The Basic SP3 Protocol was successfully applied for the range of 0.1–100 μg of protein input under constant conditions. We recommend unified amounts of beads (600 μg) and trypsin (2 μg) showing applicability of wide range of bead:protein and trypsin:protein ratios spanning from 6,000:1 to 20:1, respectively, for 0.1 μg input down to 6:1 and 1:50 for 100 μg protein input. In case of low amounts (≤10 μg) high bead:protein and trypsin:protein ratios proved to be beneficial probably promoting interactions of the proteins and beads providing better results than FASP. Application of our Basic SP3 Protocol enabled the identification of a similar number of peptides and proteins in AT-SDT lysate (for comparable protein inputs) in comparison to the more time-demanding workflows implemented by Song et al. (2018), which included phenol extraction or urea extraction/acetone precipitation from AT leaves followed by FASP digestion.

When processing large quantities of protein, SP3 beads aggregated heavily and became sticky, resulting in potential losses to inner plastic surfaces. Therefore, for applying of milligram inputs (up to 10 mg) of protein in the Large-Scale SP3 Protocol, we recommend keeping an adequate excess of beads, maintaining the working ratio of SP3 beads:protein 10:1 (w/w), and also using an increased sample volume of 0.5 ml. Sera-Mag SpeedBeads are recommended for the larger amounts of protein as they sediment on the magnet faster than the classic Sera-Mag Beads; otherwise SP3 on both types of beads is equal. For the digest, the adjusted amount of trypsin in 2 ml 50 mM AB should be used to maintain the ratio enzyme to protein 1:100.

The additional washing step with of 50 mM AB (2 ml) after collection of digestion solution was implemented because about one-tenth of the peptide amount remained on beads. The first additional supernatant was pooled with the original digest solution. After spinning at 20,000 g, preventing any potential bead carryover, peptide samples in the volatile AB buffer can be directly used for MS analysis or other follow-up processing without any additional cleanup.

## Conclusion

Four different sample preparation methods were compared for plant proteome bottom-up analysis, and two of them were eventually selected and benchmarked closely, the SP3 on carboxylated magnetic beads as the alternative to the well-established FASP method. Both workflows were tested under a wide range of protein concentrations 0.1–100 μg, 5 and 10 mg. SP3 Carboxy provided a higher number of identified protein groups and peptides for low protein inputs (≤10 μg). For higher protein inputs, both methods were comparable in all monitored features. Concerning SP3 Carboxy we described two protocol variants – first, the Basic SP3 Protocol covering range of protein input 0.1–100 μg with no need to adjust further the amount of beads, binding and rinsing conditions, and/or digestion parameters and second, the moderately modified Large-Scale Protocol which is designed for processing of milligrams of protein. Furthermore, the easy-to-use SP3 Carboxy protocol can be completed in 2 h (or in 90 min if the alkylation step is omitted), while the rather labor-intensive FASP procedure takes about 5 h and more (calculated without digestion time). Together with significantly lower costs, we assume that our proposed SP3 protocols provide a reliable tool for economical, rapid, and easily automatable single-tube preparation of plant samples for bottom-up MS-based proteomics enabling the SDS utilization without any compromise. We assume that the protocols designed for complex plant samples will be sufficiently robust also for other types of samples.

## Data Availability Statement

The datasets presented in this study can be found in online repositories. The names of the repository/repositories and accession number(s) can be found at: https://www.ebi.ac.uk/pride/archive/, PXD022688.

## Author Contributions

HK and ZZ set up the experimental design. RH and HK performed the sample preparation of AT samples. KM and DP performed LC-MS/MS analyses and data processing. HK, KM, JH, and ZZ interpreted the results. All authors contributed to manuscript writing, and have given approval to the final version of the manuscript.

### Conflict of Interest

The authors declare that the research was conducted in the absence of any commercial or financial relationships that could be construed as a potential conflict of interest.
