# Supplementary material for: SP3 Protocol for Proteomic Plant Sample Preparation Prior LC-MS/MS
Source: Front Plant Sci. 2021 Mar 10;12:635550. doi: 10.3389/fpls.2021.635550 (PMC7988192; doi:10.3389/fpls.2021.635550)
Supplement: Supplementary file 1 [file Data_Sheet_1.docx]

Supplementary Material

# Supplementary Figures and Tables

## Supplementary Tables

| **method** | **PGs** | **% of total unique PGs** |
| --- | --- | --- |
| **SP3 Carboxy** | 3324 | 89.52 |
| **FASP** | 3298 | 88.82 |
| **SP3 HILIC** | 3204 | 86.29 |
| **S-Trap** | 3305 | 89.01 |
| **combined PGs** | 2737 | 73.71 |
| **total unique PGs** | 3713 |  |

**Supplementary Table 1.** Total number of identified unique protein groups (protein FDR q < 0,01) within individual methods (PGs identified in at least one of 3 replicates; injected 2.5 µg per analysis). Combined PGs – PGs identified using all four methods; total unique PGs – sum of unique PGs over all methods.

## Supplementary Figures


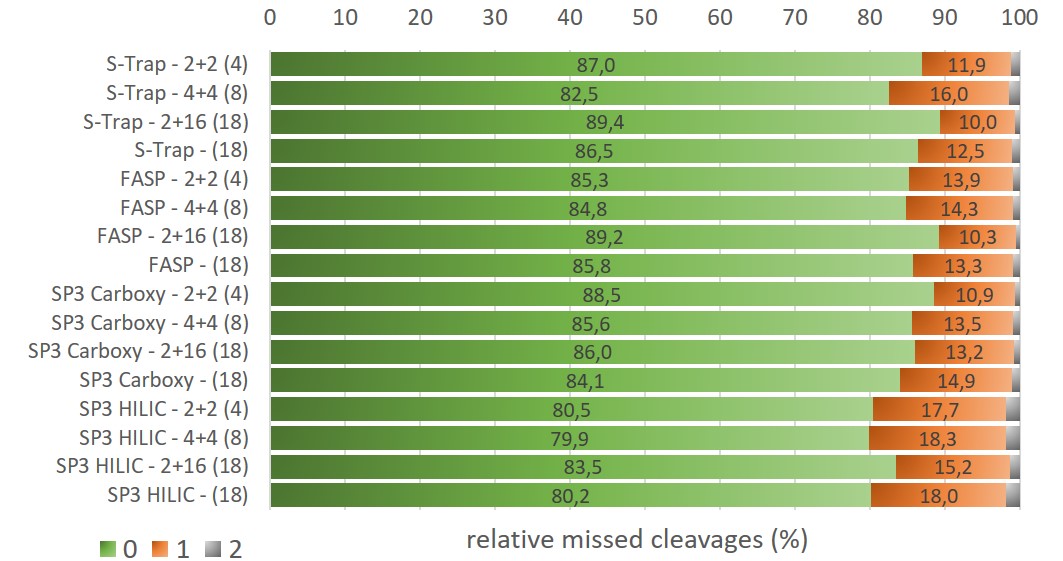


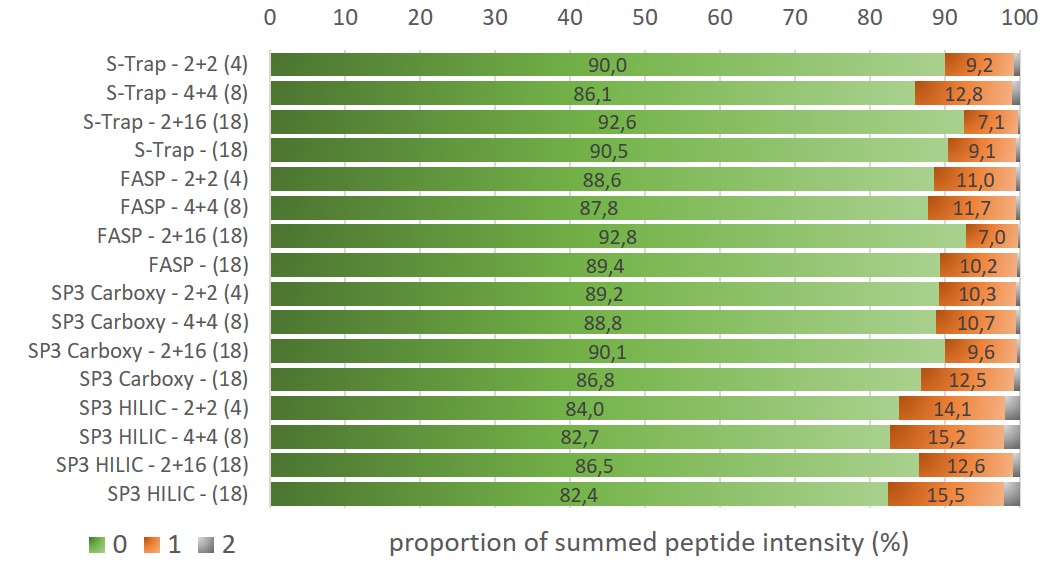


**Figure S1.** Comparison of digestion efficiency of FASP, S-trap, SP3 Carboxy and SP3 HILIC. Method comparison of missed cleavage peptide content **(upper)** and corresponding proportion of summed peptide intensity **(bottom)**. Various cleavage time and multiple trypsin addition were tested for *Arabidopsis thaliana* sample. Relative missed cleavages content and peptide intensity proportion correspond to percentage of identified peptides without any missed cleavages (green), with one (orange) and two (grey) missed cleavages. Total digestion time is displayed in brackets. Totally 12 µg of trypsin was used, 6 µg for each addition. The relative content was calculated as a mean from 3 repetitions.


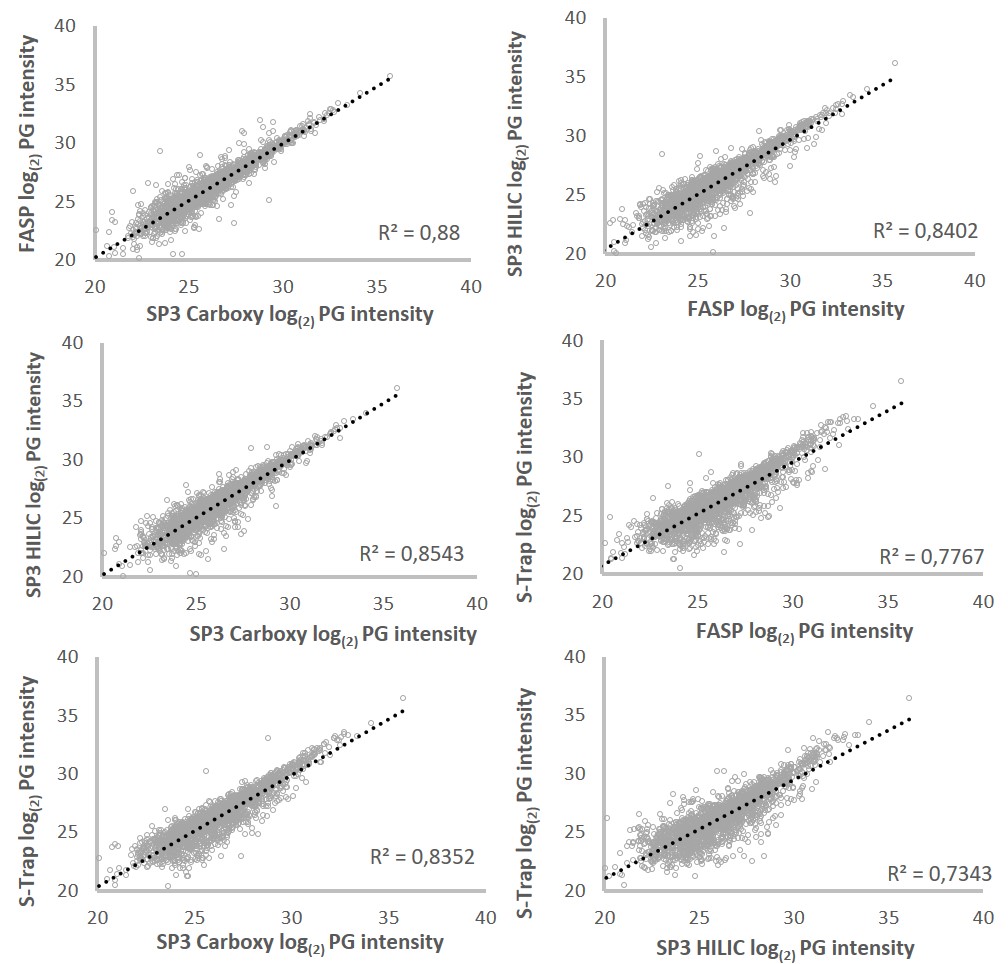


**Figure S2.** Scatter plots of SP3 Carboxy, SP3 HILIC, FASP, and S-Trap methods for *Arabidopsis thaliana* samples subjected to 18 hours trypsin digestion (protein:trypsin 10:1). Method correlation was verified by coefficient of determination (R^2^). Median intensity (3 replicates) and median normalization approach were employed to calculate protein group intensity. Data were log_(2)_ transformed.


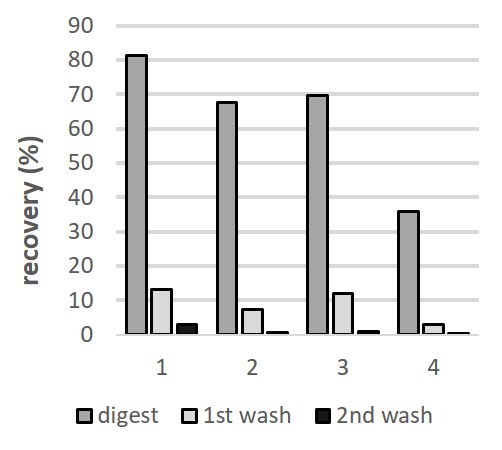


**Figure S3.** Comparison of SP3 Carboxy method performance for 10 mg input amount of *A. thaliana* samples. Obtained recoveries in digestion solution and in subsequent washes for individual conditions (relative recoveries to digest amount).


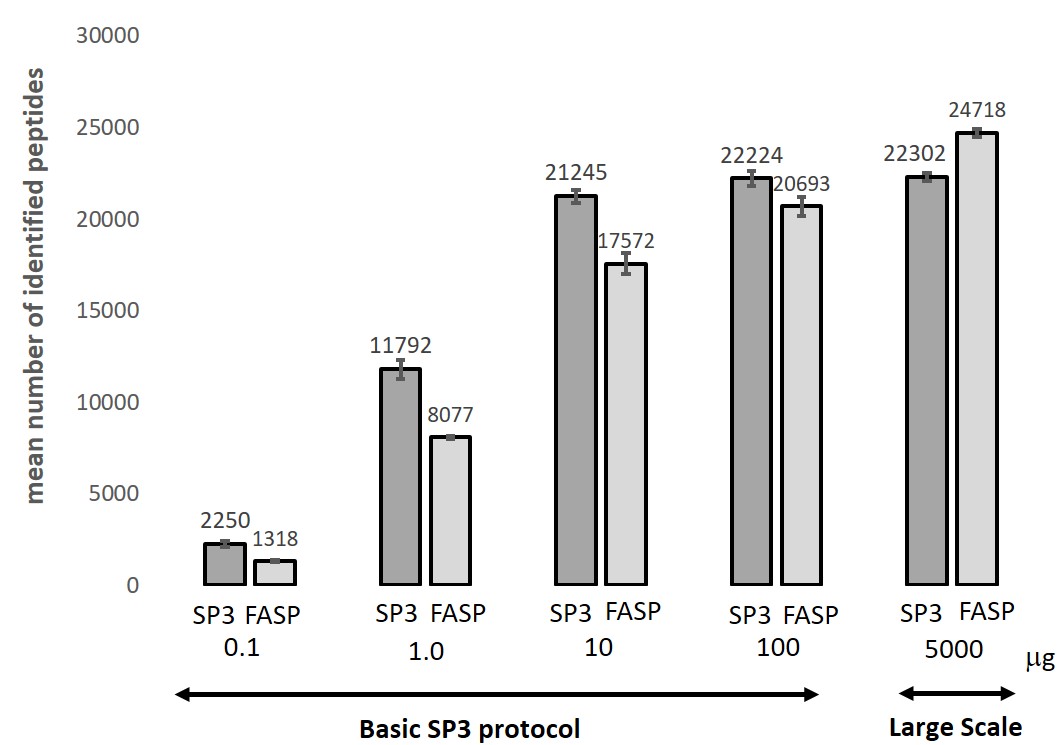


**Figure S4.** Comparison of mean number of identified peptides for SP3 Carboxy and FASP methods over tested protein load range. Standard deviation was applied for error bars calculation. Data were calculated as the average value from 3 or 5 (100 µg) replicates. The only half of the final digested sample was injected for 0.1 µg and 1 µg input amounts to prevent loss of sample due to technical issues. In case of higher sample loads (≥ 10 µg), we injected about 2 µg of tryptic peptide mixture.


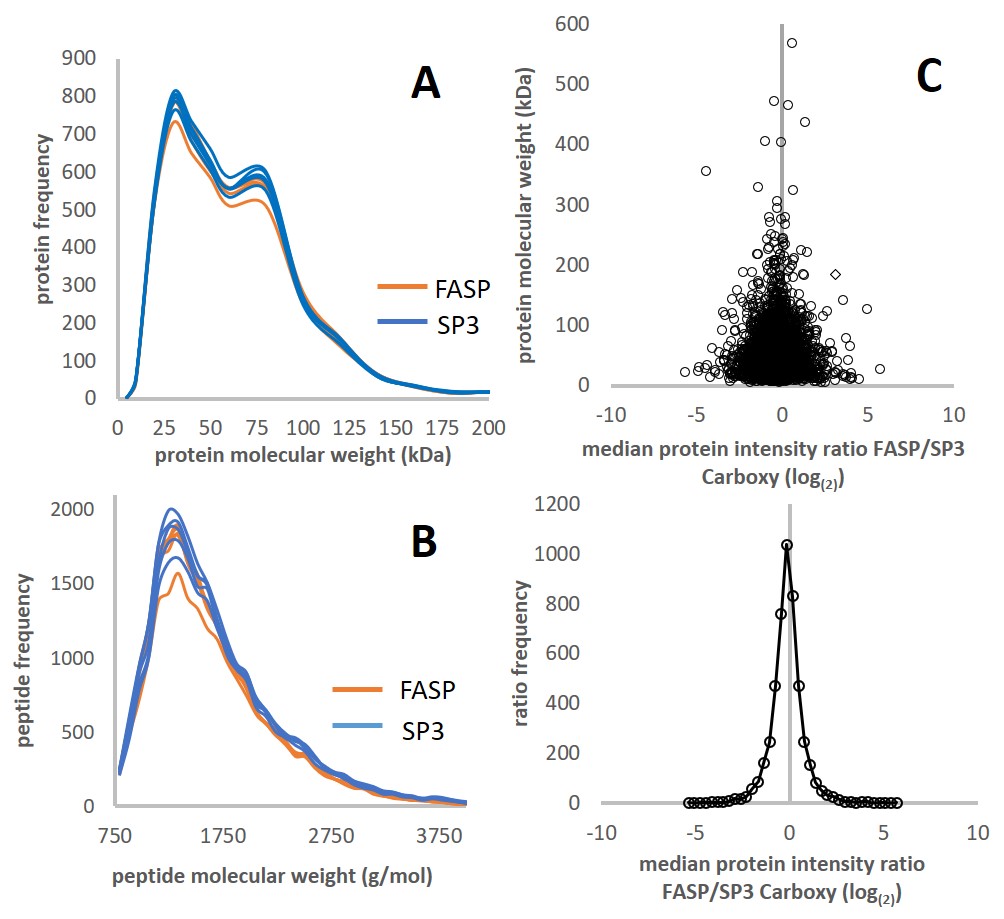


**Figure S5.** Molecular weight distribution of proteins **(A)** and peptides **(B)** for FASP and SP3 Carboxy methods. Median protein intensity FASP/SP3 Carboxy ratios including ratio frequency versus protein molecular weight **(C)**. Pentaplicate measurements were done for each method at 100 µg protein load.


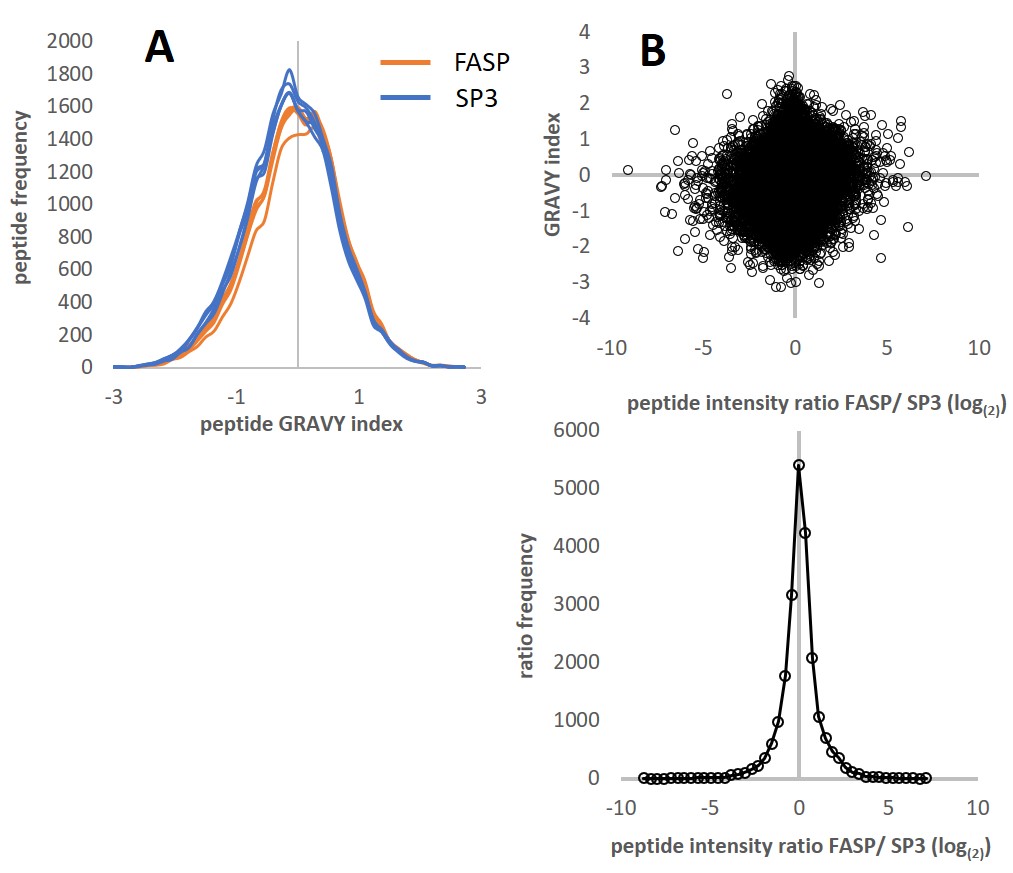


**Figure S6.** Peptide GRAVY index distribution **(A)** and peptide intensity FASP/SP3 Carboxy ratios including ratio frequency versus GRAVY index **(B)**. Pentaplicate measurements were done for both methods at 100 µg protein load.


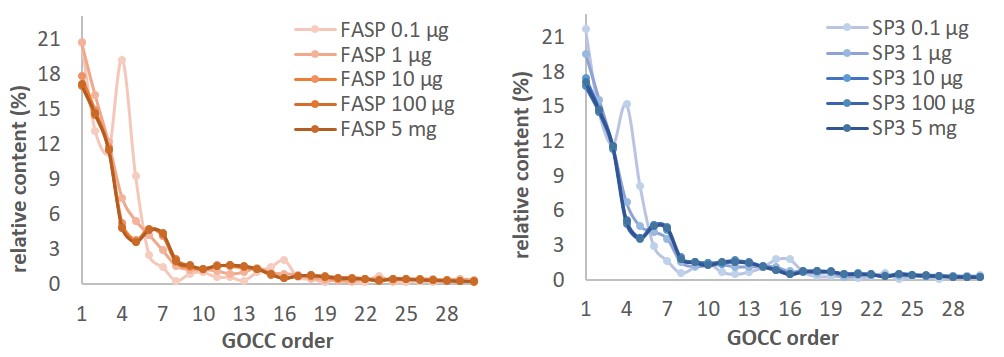


| order | GOCC name | order | GOCC name |
| --- | --- | --- | --- |
| **1** | chloroplast | **16** | cytosolic ribosome |
| **2** | cytosol | **17** | membrane |
| **3** | cytoplasm | **18** | peroxisome |
| **4** | apoplast | **19** | endoplasmic reticulum membrane |
| **5** | cell wall | **20** | nucleolus |
| **6** | endoplasmic reticulum | **21** | chloroplast envelope |
| **7** | integral component of membrane | **22** | vacuole |
| **8** | extracellular region | **23** | cytosolic small ribosomal subunit |
| **9** | mitochondrion | **24** | catalytic step 2 spliceosome |
| **10** | Golgi apparatus | **25** | mitochondrial matrix |
| **11** | plasma membrane | **26** | anchored component of plasma membrane |
| **12** | nucleus | **27** | chloroplast stroma |
| **13** | endosome | **28** | chloroplast thylakoid membrane |
| **14** | anchored component of membrane | **29** | extrinsic component of membrane |
| **15** | cytosolic large ribosomal subunit | **30** | COPI vesicle coat |

**Figure S7.** Gene ontology analysis of the proteins identified for FASP (left) and SP3 Carboxy (right) methods. Classification according to cellular components was done via Perseus software. Most abundant thirty protein classes were selected and displayed.

# Supplementary material – protocols

## Materials

1. SDT buffer: 4% sodium dodecyl sulfate SDS; 0.1M dithiothreitol; 0.1M Tris-HCl, pH 7.6

2. Sera-Mag Carboxylate-Modified Beads (GE Life Sciences), hydrophilic solids 50 µg/µL (cat. no. 24152105050250), and hydrophobic solids 50 µg/µL (cat. no. 44152105050250)

3. Sera-Mag SpeedBeads Carboxylate-Modified (GE Life Sciences), hydrophilic solids 50 µg/µL (cat. no. 45152105050250), and hydrophobic solids, 50 µg/µL (cat. no. 65152105050250)

4. Iodoacetamide IAA 100 mM stock (keep in the dark)

5. Dithiothreitol DTT 100 mM stock

6. 100% ethanol

7. 50 mM ammonium bicarbonate

8. Trypsin, Sequencing Grade (Promega)

9. ThermoMixer C with ThermoTop (Eppendorf)

10. Ultrasonic Processor UP100H (Hielscher)

11. Pure Proteome Magnetic Stand (Merck, cat.no. LSKMAGS08)

12. Bath sonicator

13. S-Trap Mini Spin Columns (ProtiFi)

14. Vacuum manifold VM20 (Merck)

15. Magnetic multi-mode HILIC microparticles MagReSyn HILIC (ReSyn Biosciences)

## SP3 Carboxy protocols:

## 2.2.1 Basic Protocol: SP3 processing of protein load 0.1 – 100 µg

The proposed workﬂow is compatible with a broad range of protein input; therefore, the protocol should not be scaled up regarding beads amount and concentration of trypsin. The recommended working volumes should be used.

1. Set the ThermoMixer to 95°C and 1 000 rpm mixing. Extract proteins from frozen plant powder in the hot SDT buffer for 2 h. Spin the tubes for 10 min at 20 000g and RT.

2. Sonicate the extract with a probe: 5x 30 s pulses, 5x 30 s pulses pause, cool the sample during the procedure.

3. Optional: Measure protein concentration by the tryptophan fluorescence assay.

4. Precool the Thermomixer to 24°C and set for 600 rpm. Add 7.5 μL of IAA stock to 30 μL of the sample and incubate in a final 20 mM IAA in the dark for 30 min.

5. In the meantime, wash the beads and aliquot them.

6. **Beads wash**. Remove beads in original bottles from the fridge and flick them at RT ensuring complete homogenization of beads in the solution. Take 200 µL (10 mg of beads) of each original bead stock and combine them in a single 2 mL transparent tube (conical bottom), making a sufficient amount of beads for about 30 samples. On the magnetic rack pellet the beads for 1 min, remove the supernatant by pipette. Off the rack, reconstitute the beads in 1 mL of water, and pipette-mix. The prepared stock of beads 20 μg/μL can be stored at 4°C for one month.

7. Add 2 μL of DTT stock to the whole volume of the alkylated sample to the DTT final concentration 5 mM. The quenched sample can be stored at -20 °C.

8. Add 30 μL of the SP3 bead stock to the whole volume of the alkylated/quenched protein sample. Pipette-mix to homogenize the beads and lysate.

9. **Binding**. Add 70 μL of 100% ethanol to the suspension to achieve 50% ﬁnal ethanol concentration. Pipette-mix brieﬂy to homogenize the beads, lysate, and ethanol. Minimize post pipetting. Beads may clump and become sticky. Incubate tubes in the ThermoMixer for 10 min at 24°C and 1 000 rpm. On the magnetic rack pellet the beads for 2 min, remove and discard the supernatant by pipette, do not disrupt the beads.

10. **Rinsing.** Off magnetic rack add 140 μL of fresh 80% ethanol immediately.

Gently pipette-mix. Incubate on the magnetic rack for 2 minutes at RT. Remove and discard the supernatant. Repeat step 10 three further times. On the magnetic rack, always incubate for 2 minutes at RT, remove and discard the supernatant.

11. During the last 5th wash, add off the magnetic rack 140 μL of 80% ethanol. Gently pipette and transfer the whole volume of the suspension into a new 2 mL tube. Remove all liquid by pipette and continue with the digestion.

12. **Digestion**. Make an appropriate volume of new stock of trypsin 0.1 μg/μL in 50 mM AB. Off rack, add to each sample 20 μL of trypsin stock (2 μg) and 40 μL 50 mM AB, making the final digestion volume 60 μL. Avoid pipette mixing! Instead, sonicate the tubes in a water bath for 30 s (use a floating tray).

13. Incubate samples in the ThermoMixer with ThermoTop for 18 h at 37°C and 1 000 rpm.

14. On the magnetic rack pellet the beads for 2 min, recover the supernatant into 0.5 mL tube.

15. Off the magnetic rack pipette-mix with 60 μL of 50 mM AB to elute remaining peptides. Spin the tubes at 20 000g for 1 minute.

16. On the magnetic rack, pellet the beads for 2 min, recover the supernatant and pool it with the first part of the eluate.

17. Spin the whole supernatant (120 μL) at 20 000g for 10 min (remaining beads could clog the chromatographic column).

18. Optional: Measure peptide concentration by the tryptophan fluorescence assay.

19. Optional: Reducing of sample volume, e.g. on the SpeedVac.

20. Just before LC-MS/MS, spin the peptide sample again at 20 000g for 10 min.

### 2.2.2 Large-Scale Protocol: SP3 processing of protein load 5 mg

The proposed workflow is compatible with milligram quantities of protein (up to 10 mg) using 500 μL of sample volume. Always keep the ratio beads:protein 10:1. For the digest, the adjusted amount of trypsin in 2 mL 50 mM AB should be used to maintain the ratio enzyme to protein 1:100. The recommended working volumes should be used.

1. Set the ThermoMixer to 95°C and 1 000 rpm mixing. Extract proteins from frozen plant powder in the hot SDT buffer for 2 h. Spin the tubes for 10 min at 20 000g and RT.

2. Sonicate the extract with a probe: 5x 30 s pulses, 5x 30 s pulses pause, cool the sample during the procedure.

3. Optional: Measure protein concentration by the tryptophan fluorescence assay.

4. Precool the Thermomixer to 24°C and set for 600 rpm. Add 125 μL of IAA stock to 500 μL of the sample and incubate in a final 20 mM IAA in the dark for 30 min.

5. In the meantime, wash the beads and aliquot them.

6. **SpeedBeads wash**. Remove beads in original bottles from the fridge and flick them at RT ensuring complete homogenization of beads in the solution. For 5 mg of protein, take 500 µL (25 mg of beads) of each original bead stock and combine them in a single 5 mL transparent tube, making a sufficient amount of beads for one sample. On the magnetic rack pellet the beads for 5 min, remove the supernatant by pipette. Off the rack, reconstitute the beads in 5 mL of water, and pipette-mix. Repeat the wash four more times. The prepared stock of beads can be stored at 4°C for one month.

7. Add 33 μL of DTT stock to the whole volume of the alkylated sample to the DTT final concentration 5 mM. The quenched sample can be stored at -20°C indefinitely.

8. On the rack, pellet the washed beads for 5 min and remove water by pipette.

9. Add the whole volume (658 μL) of the alkylated/quenched protein sample to the beads. Pipette-mix to homogenize the beads and lysate.

10. **Binding.** Add 658 μL of 100% ethanol to the suspension to achieve 50% ﬁnal ethanol concentration. Pipette-mix brieﬂy to homogenize the beads, lysate, and ethanol. Minimize post pipetting; beads may clump and become sticky. Incubate tubes in the ThermoMixer for 10 min at 24°C and 1 000 rpm. On the magnetic rack pellet the beads for 5 min, remove and discard the supernatant by pipette, do not disrupt the beads.

11. **Rinsing.** Off magnetic rack add 4 mL of fresh 80% ethanol immediately.

Gently pipette-mix. Incubate on the magnetic rack for 5 minutes at RT. Remove and discard the supernatant. Repeat step 11 four further times. On the magnetic rack, always incubate for 5 minutes at RT, remove and discard the supernatant.

12. During the last 6th wash, add off magnetic rack 4 mL of 80% ethanol. Gently pipette-mix and transfer the whole volume of the suspension into a new 5 mL tube. Incubate on the rack for 5 min, remove all liquid by pipette and continue with the digest.

13. **Digestion.** Make an appropriate volume of new stock of trypsin 0.1 μg/μL in 50 mM AB. Off rack add to each sample 500 μL of trypsin stock (50 μg) and 1.5 mL 50 mM AB, making the final digestion volume 2 mL. Avoid pipette mixing! Instead, sonicate the tubes in a water bath for 5 min (use a floating tray).

14. Incubate samples in the ThermoMixer with ThermoTop for 18 h at 37°C and 1 000 rpm.

15. On the magnetic rack, pellet the beads for 5 min and recover the supernatant into the 5 mL tube.

16. Off the magnetic rack pipette-mix with 2 mL of 50 mM AB to elute remaining peptides. Spin the tubes at 20 000g for 1 minute.

17. On the magnetic rack, pellet the beads for 5 min, recover the supernatant, and pool it with the first part of the eluate.

18. Spin the whole volume of supernatant (2x 2 mL) at 20 000g for 10 min (remaining beads could clog the chromatographic column).

19. Optional: Measure peptide concentration by the tryptophan fluorescence assay.

20. Just before LC-MS/MS, spin the peptide sample again at 20 000g for 10 min.

### 2.2.3 Important Notes for SP3 Carboxy workflows

**Changing tubes** between washes sorted out the SDS contamination because residual traces of SDS were found on tube walls rather than carried over with the beads (S. Stoychev, ReSyn Biosciences - personal communication, May 2019).

**DNA shearing** DNA and RNA can be captured by the carboxylate-modified beads as well, resulting in higher stickiness of the beads. To prevent a detrimental effect of material loss on the inner plastic surfaces, preliminary shearing steps are recommended before SP3, such as sonication, bead beating, or treatment with benzonase (Hughes 2019). The resulting sample can be subsequently processed by the SP3 for the protein cleanup.

**Speed Beads for the Large-Scale Protocol.** Sedimentation of SpeedBeads on the magnet was faster than sedimentation of Sera-Mag, otherwise SP3 on both types of beads was equal. Milligram quantities of protein (up to 10 mg) can be processed by the Large-Scale Protocol using 500 µL of the sample. For the digest, the adjusted amount of trypsin in 2 mL 50 mM AB should be used to maintain the ratio enzyme to protein 1:100.

**Ethanol usage.** As a general rule, the binding step was designed to proceed at the working ethanol concentration of 50 % and to continue with all the rinsing steps in 80 % ethanol.

## 2.3 SP3 HILIC

AT lysis is descibed in the manuscript Methods – Protein extraction. Otherwise the **MagReSyn HILIC beads** (ReSynBio) were used according to the RAPOBD protocol from <https://resynbio.com/wp-content/uploads/2019/12/HILIC_RAPOBD.pdf> with minor changes - the enzyme: protein ratio was 1:25 instead of 1:20 to keep the ratio the same as for the SP3 carboxy protocol. Briefly, washed MagReSyn® HILIC microparticles were equilibrated in the equilibration buffer (15% ACN, 100 mM ammonium acetate pH 4.5) three times. After removal of this buffer, the protein sample containing 60 μg total protein, was mixed with an equal volume of 2 x binding buffer (30% ACN in 200 mM ammonium acetate, pH 4.5). Proteins were allowed to bind to microparticles for 30 min. After separation of the supernatant, the beads were washed with 200 μl wash buffer (95% ACN), and mixed by gentle vortexing for 1 min. On the magnetic separator recovered beads were resuspended in 200 μl wash buffer (95% ACN), the microparticle suspension was transferred to a new tube to avoid potential carryover of detergent from the tube. Recovered microparticles were resuspended in 60 μl of digestion buffer (50 mM ammonium bicarbonate), containing a suitable amount of trypsin. Samples were incubated at 37°C for the desired period of time, gently mixed at 300 rpm in the Thermomixer. The microparticles were separated on the magnetic separator; supernatant containing peptides was transferred to a 0.5 ml Eppendorf tube and applied to a magnetic separator to remove any beads that may have been carried over. The protein content in the supernatant was measured by tryptophan assay.

**2.4 S-Trap**

AT lysis is descibed in the manuscript Methods – Protein extraction. Otherwise the **S-Trap Mini Spin Columns** (ProtiFi) were used according to the *S-Trap Mini Spin Column Digestion Protocol 3.6* with minor changes - the amount of protein applied on the column (through a vaccuum manifold) was 60 μg since the manufacturer originally recommended amounts up to 100 μg for the S-Trap Mini (<https://john-wilson-ymlv.squarespace.com/>). Later on, the manufacturer´s guidelines were changed: S-Trap **Mini** columns were recommended for 100 – 300 μg of protein and S-Trap Micro for amounts lower than 100 μg. The Protifi web page does not show the protocol used in our study any more. Here is the detailed description:

**
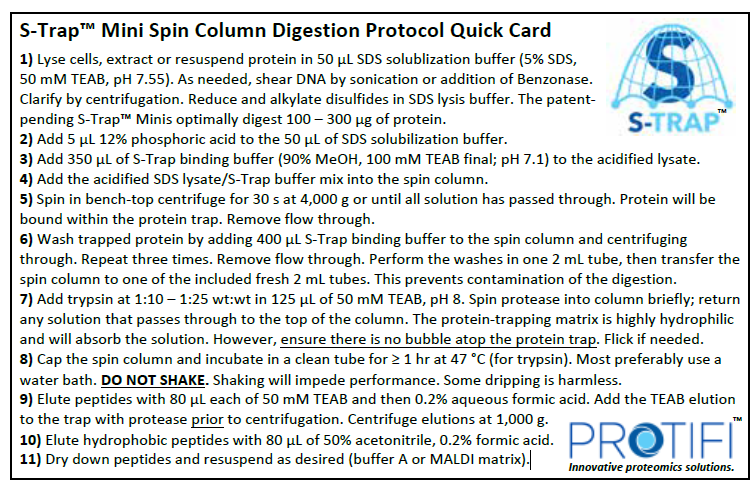
**
